# Medical and Physical Intelligence

**Published:** 1806-08

**Authors:** 


### MEDICAL AND PHYSICAL INTELLIGENCE.

*Extract of a Letter from Capt.* Macnamara, *in the Hon. East India Company's Service, to the Editors of the Medical and Physical Journal, dated Oct.* 24, 1805.

"During my residence at Cheltenham, this autumn, a circumstance occurred to me that, as thousands may be benefited by its being made public, I request the favour of your rendering it generally known through the extensive circulation of your publication.

"I was proceeding towards the Well on the morning after my arrival, accompanied by Mrs. Macnamara, when we met a woman carrying a child, whose face was covered with an ill-looking erup-tion, tion, which bore so strong a resemblance to the Small-pox, that it alarmed us considerably, as we had our only child with us, about six weeks old, who had never been inoculated. Our apprehensions led us to enquire of the woman the cause of this shocking appearance, when she informed us, *That the child had been inoculated about twelve months back with the Cow pox, and had, in consequence, been in that condition ever since.*—Having intended to consult Dr. Jenner respecting the inoculating of my own child with vaccine matter, I was so far staggered by this circumstance, as almost to give up the idea of it, when fortunately happening to communicate my fears to a friend, an eminent physician in London, he gave me so very favourable an account of the new practice, his own son having undergone it without a moment's uneasiness or difficulty, that I immediately waited on Dr. Jenner, and frankly avowed the whole to him. He delivered himself so clearly and satisfactorily on the subject of vaccination, that I not only resolved on having my child inoculated, but felt it incumbent on me to trace what I now was fully persuaded would prove to be either a mistake or a gross misrepresentation. I therefore waited on the mother* of the child, accompanied by Mr. Liddle, a medical friend, when she told us *the child had never been inoculated at all*, but that she intended taking it to Dr. Jenner for that purpose, since the Cow-pox inoculation had entirely cured another of her children,† who had been afflicted with a similar eruption.

"The reflection that, had we quitted Cheltenham without any investigation of this affair, we might, innocently, have been the instruments of propagating a most injurious falsehood, persuaded both my friends and myself of the absolute necessity of publishing the case as it really is. I should not only have been convinced myself that this was an instance of Small-pox, or something as bad after Cow-pox; but should have publicly declared *the fact* as happening within my own observation and knowledge.—Without dwelling on the consequence this might have been to others, I cannot but remark that my child might have suffered disfiguration, if not death, from the Small-pox; whereas, on the contrary, by having been inoculated with the vaccine matter, she is now rendered proof against that loathsome disease, without having ever suffered a moment's uneasiness, or having the least mark on her person, except that left on the arm by the vaccine pock.

"Under the full assurance of all agreeing that so valuable a discovery and important benefit to mankind ought not to be checked by similar ignorance or malevolence, I trust you will allow the circumstance a place in your widely-circulating publication."

An

* The woman whom we first saw with the child was its nurse occasionally.

† We understand this child, of whom the nurse made the false and wicked report, was afterwards vaccinated, and the skin, as in the former instance, became almost immediately after perfectly free from eruptions. Such instances are become familiar to us. Ed.

An important fact with regard to the theory of electricity, has recently been discovered by M. Bienvenu. By varying his experiments he has found, in contradiction to the received opinion, that glass and rosin produce the same kind of electricity, and that the difference depends upon the rubbers. With a cat's skin he electrizes an electrophorus of rosin, which manifests negative electricity; an electrophorus made of a piece of glass, and rubbed with a cat's skin, manifests exactly the same kind of electricity as that of rosin. This experiment proves, that if the conductor of an electrical machine constantly gives positive electricity, the reason lies in the morocco cushions, which possess the property of developing the electricity of glass, which, received on the conductor, communicates to it a positive electricity. To prove this, he substitutes cushions of cat's skin in their stead; the glass is then negatively electrized, and the conductor furnishing it with the electricity it has lost, manifests a negative electricity.

---

The following receipt for keeping flies out of apartments and stables, and driving them away from horses was sold in a sealed cover at the Leipsic Michaelmas fair, at a high price, and had a very extensive sale. Put into an earthen pot half a pound of cantharides, an ounce and a half of grain seed; mother-wort, sassafras, root of St. John's wort, and spirit of ants, of each half an ounce; a quarter of an ounce of orpiment, a good handful of savin, the whole cut small or reduced to powder: close the pot hermetically, luting the interstices of the lid with flour-paste. After the contents of the pot have boiled sufficiently, take it from the fire, and let it stand 24 hours in a cool place: then uncover the pot, and with a feather smear the frames of the windows and doors, both of apartments and stables, from which you are desirous of keeping the flies. A single coat is sufficient for the whole season; but if the rain should chance to take it off, care must be taken to renew it. The smell of this preparation, which is scarcely perceptible to man, is so insupportable to flies that there is not a single instance of one having entered by an open window or door to which this liquid has been applied. To keep them away from horses, it is sufficient to besmear the harness, the girth, or the saddle, with this liquid.

---

The following decree has been issued by his Catholic Majesty the King of Spain, on the occasion of some experiments made at Carthagena, with respect to the efficacy of anti-contagious fumigations. "Don F. de Borja, commander in chief at Carthagena, having made known to the King, in different Reports, the important services performed by Don Michel Cabanellas, during the prevalence of the contagious distemper which raged in that place, his Catholic Majesty was particularly struck with the importance of the experiment made by him in one of the hospitals of the said city, city, where he shut himself up with fifty persons, in order to prove the efficacy of the acid fumigations, and actually slept with his companions, including two of his own children, in the beds where many patients had recently fallen victims to this terrible disease, without employing any other preservative means than the mineral acid fumigations, as directed by M. Guyton. His Catholic Majesty, moreover, learned with the most unfeigned satisfaction, that the result of the experiment was so fortunate, that the fifty-one persons, after having been strictly confined in this lazaretto, had come out of it in a state of perfect health. In consequence, and in order to afford a proof of his royal munificence, his Catholic Majesty has remitted to each of the galley slaves who voluntarily submitted to this experiment (not having previously undergone an attack of the yellow fever) one year of the time they were sentenced to remain in chains; and he farther caused his approbation of their conduct to be notified to them by his captain-general. To Don Michel Cabanellas his Catholic Majesty grants the title and honours of physician to his Majesty's household, with an annual salary of 24,000 reals, to be paid monthly from the funds of the community of Carthagena; at the same time has conferred on him a right of voting in the municipal body of that city, in the same manner as if he had been a natural born citizen. The king, besides, charges himself with providing for his two children, whose lives, like his own, were exposed for the interest of the state and of humanity."

---

*An infallible Remedy for stopping Hæmorrhages from the Nose.*—This remedy has been universally in use for more than a hundred years in the province of Frisia, but was kept a profound secret until M. Tjalingii, apothecary, at Amsterdam, made its composition public, which is as follows:

℞. Sacchari Saturni unciam unam, vitrioli martis unciam semis, seorsim terantur in mortaris vitrio, adde spiritus vini uncias octo. M. Of this composition, young people from ten to twelve years of age, are to take 10 or 12 drops; patients under twenty years, 14 or 15 drops; and grown persons 20 drops, four times each, in a spoon full of wine or brandy. He himself has made some very interesting trials in the most obstinate cases, with the greatest success. By analogy, he recommends the same medicine for the cure of hæmorrhages of all kinds, particularly of the uterus, which often prove very tedious.

---

Mr. Home has furnished to the Royal Society an interesting paper on the comparative anatomy and physiology of the camel, particularly on its stomachs and water-bags, in which it can retain a quantity of water sufficient to support itself for several weeks.

---

Mr. Davy has discovered that the acid, which exists in minute quantities in the wavellite (the new fossil from Barnstable) is the fluoric fluoric acid, in such a peculiar state of combination as not to be rendered sensible by sulphuric acid.

M. de Threba, superintendant of the mines of Freyberg, and Professor Lampadius, have lately ascertained, by repeated experiments, the relative temperature of the internal parts of the earth. Having placed, at different depths in the mines, two of Reaumur's thermometers, and compared them twice every day with another exposed in open air, they found that whatever difference of temperature prevailed above ground, one of the two thermometers placed in the mines uniformly indicated 12° above zero, and the other $9\frac{1}{2}$°.

A letter has recently been received from M. Rehmann, the physician in the suite of the Russian embassy to China, dated Kiachta on the frontiers of China, October 14, 1805; in which he says, that he has vaccinated a great number of the children of the Mogols.

The first fasciculus of the long expected Flora Græca of the late Professor Sibthorp, edited by Dr. Smith, will make its appearance in a few days. It will consist of 50 plates, beautifully coloured, with descriptive letter-press. This splendid work will form, when compleated, ten volumes in folio, containing one thousand figures, executed by Sowerby from the masterly drawings of Mr. Ferdinand Bauer.

Mr. Astley Cooper will in a few weeks publish the concluding part of his great work on Hernia.

